# Systematic review and meta-analysis of mass spectrometry proteomics applied to ocular fluids to assess potential biomarkers of age-related macular degeneration

**DOI:** 10.1186/s12886-023-03237-0

**Published:** 2023-12-12

**Authors:** Hanmu Guo, Jianqing Li, Peirong Lu

**Affiliations:** Department of Ophthalmology, The First Affiliated Hospital of Soochow University, Suzhou, China

**Keywords:** Age-related macular degeneration, Proteomics, Aqueous, Vitreous

## Abstract

**Background:**

Age-related macular degeneration (AMD) is a significant cause of severe vision loss. The main purpose of this study was to identify mass spectrometry proteomics-based potential biomarkers of AMD that contribute to understanding the mechanisms of disease and aiding in early diagnosis.

**Methods:**

This study retrieved studies that aim to detect differences relate to proteomics in AMD patients and healthy control groups by mass spectrometry (MS) proteomics approaches. The search process was accord with PRISMA guidelines (PROSPERO database: CRD42023388093). Gene Ontology (GO) analysis and Kyoto Encyclopedia of Genes and Genomes Pathway Analysis (KEGG) were performed on differentially expressed proteins (DEPs) in the included articles using the DAVID database. DEPs were included in a meta-analysis when their effect size could be computed in at least two research studies. The effect size of measured proteins was transformed to the log2-fold change. Protein‒protein interaction (PPI) analysis was conducted on proteins that were statistically significant in the meta-analysis using the String online database.

**Results:**

Eleven studies fulfilled the inclusion criteria, and 161 DEPs were identified. The GO analysis showed that AMD is significantly related to proteolysis, extracellular exosome and protein binding. In KEGG, the most significant pathway was the complement and coagulation cascades. Meta-analysis results suggested that eight proteins were statistically significant, and according to PPI results, the most significant four proteins were serotransferrin (TF), apolipoprotein A1 (APOA1), complement C3 (C3) and lipocalin-1 (LCN1).

**Conclusions:**

Four possible biomarkers, TF, APOA1, C3 and LCN1, were found to be significant in the pathogenesis of AMD and need to be further validated. Further studies should be performed to evaluate diagnostic and therapeutic value of these proteins.

**Supplementary Information:**

The online version contains supplementary material available at 10.1186/s12886-023-03237-0.

## Introduction

Age-related macular degeneration, which accounts for 6–9% of legal blindness globally, is the leading cause of severe vision loss among individuals over 55 years of age in developed countries [1, 2]. Despite differences in the prevalence of AMD between eastern and western countries [3], the aging of the global population indicates an inevitable surge in the absolute number of AMD patients worldwide, with a projected increase from 196 million in 2020 to 288 million by 2040 [1].

Although there are powerful diagnostic tools such as optical coherence tomography and fundus autofluorescence imaging for AMD, the diagnosis of AMD may be delayed. One of the main challenges lies in the fact that early AMD is often asymptomatic [4]. Additionally, if only one eye is affected, symptoms may not be apparent until the visual function of the other eye is compromised. Late-stage AMD is characterized by geographic atrophy (GA) and/or neovascular AMD (nvAMD) [5]. GA is manifested as retinal pigment epithelial cells lost, overlying photoreceptors, and underlying choroidal capillaries, which does not involve blood or serum leakage [6]. Unlike GA, nvAMD is characterized by choroidal neovascularization complex which involve blood or serum leakage [4]. In the process of wet AMD, overexpression of VEGF is a crucial risk factor [7]. Meanwhile, several mechanisms are involved in both dry and wet AMD, including RPE cell senescence, oxidative stress, lipid metabolism, inflammation and immunity [8]. As advanced forms of AMD, GA and nvAMD can cause loss of central visual acuity, leading to severe and permanent visual impairment and ultimately resulting in legal blindness, which has a major impact on quality of life and functional independence [4]. In recent years, significant achievements have been made in the therapeutic strategies of nvAMD [9], such as the availability of several anti-VEGF agents, including ranibizumab [10] and aflibercept.[11] However, there is still a lack of effective strategies to prevent photoreceptor loss in the context of de novo development and enlargement of GA. Moreover, the treatment effect of AMD patients is not optimistic, primarily due to the treatment burden or potential final outcomes such as atrophy and fibrosis [12]. Hence, there is an urgent need for ongoing efforts to reduce treatment burden and enhance existing treatment options.

The etiology and pathogenesis of AMD remain unclarified. Given that biomarkers can reveal the dysregulation of molecular expression profiles, the identification of biomarkers holds great significance and provides insight for the early diagnosis, mechanism and treatment of AMD. Intraocular fluid has been proven to be suitable for the evaluation of relevant biomarkers for posterior segment disorders [13]. In recent years, many studies have demonstrated molecular differences in aqueous humor (AH) or vitreous humor (VH) between AMD patients and controls [14–16]. These findings have made significant contributions toward the identification of potential biomarkers and have shed light on the underlying mechanisms involved in AMD.

Proteomics is a powerful platform for studying both single proteins and complex protein samples [17]. In recent years, MS technologies have become an effective tool to discover biomarker, and quantitative MS proteomics such as liquid chromatography combined with tandem MS (LC‒MS/MS) approaches show fine specificity and sensitivity [18], which can detect the composition, structure, and function of proteins. In recent decades, with advancements in MS, multiple proteins have been discovered in AH and VH samples of AMD patients, which significantly contribute to uncovering potential biomarkers and shedding light on the underlying mechanisms of AMD.

While significant progress has been made in the field of AMD biomarker research using proteomic techniques, there is still a lack of well-established and validated biomarkers for AMD. Additionally, the precise mechanisms underlying AMD development and progression remain incompletely understood. This article provides a comprehensive summary of the literature on the analysis of AH and VH samples from AMD patients using MS proteomics. The objective of the study was to conduct a meta-analysis to identify potential biomarkers of AMD that could shed light on the disease's underlying mechanisms and aid in early diagnosis.

## Method

This study accorded PRISMA guidelines and registered in the PROSPERO database (CRD42021274183). Studies were included in this systematic review if they match the search keywords.

### Search strategy

**Fig. 1:**
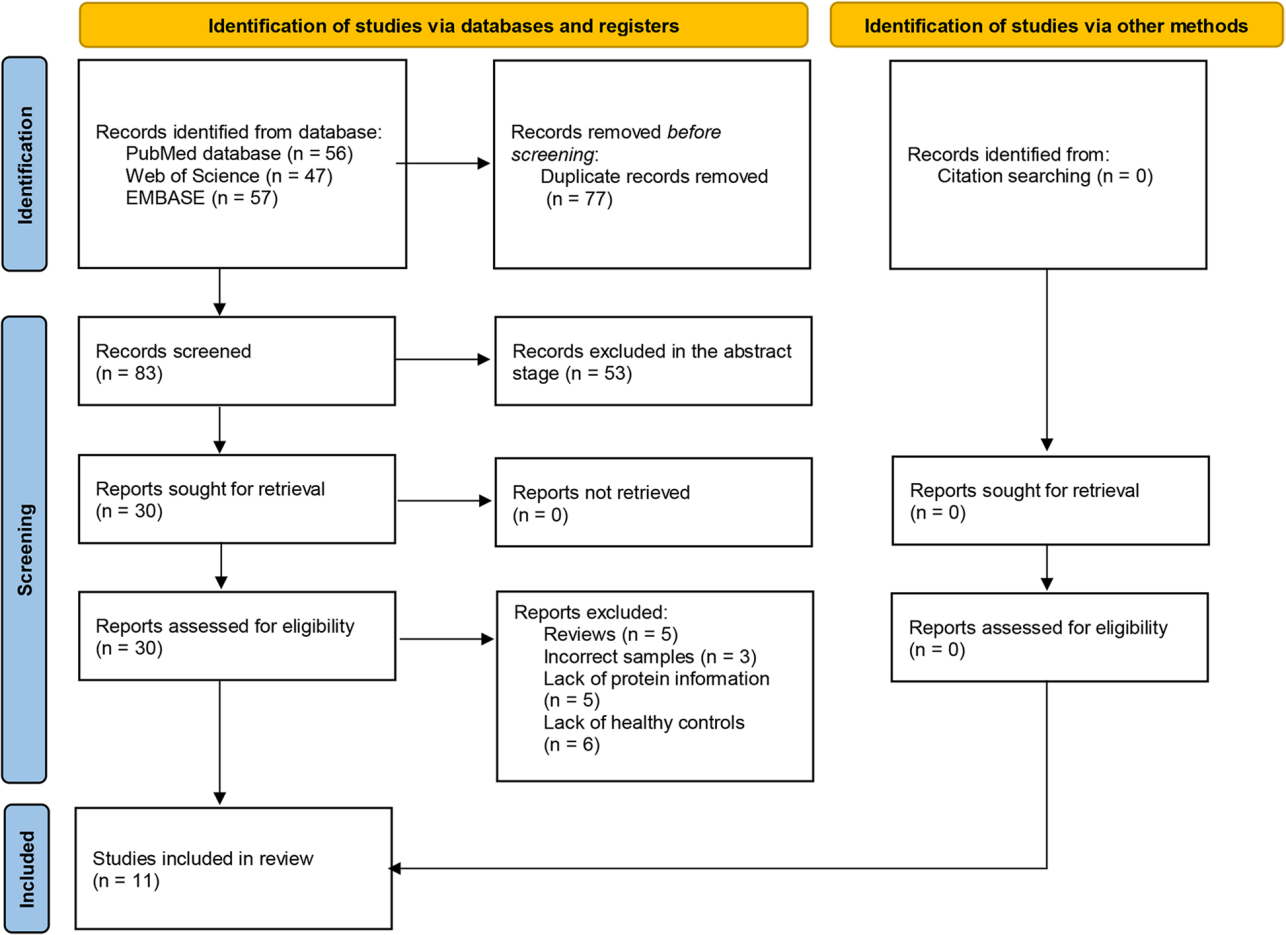
Flow chart of the selection process of the studies included in this systematic review of intraocular fluid mass spectrometry-based proteomics in age-related macular degeneration, following PRISMA 2020

The researchers conducted a comprehensive search for relevant studies through three independent databases: PUBMED, Web of Science (WOS) and EMBASE. Keywords of search process were used the MeSH or Emtree terms as well as free words, until March 2023: [(“age-related macular degeneration” OR “AMD”) AND (“aqueous humor” OR “vitreous Humor”)] AND “proteomics”. Researchers also screened references of relevant studies. The process to select the included studies in this study, which followed PRISMA 2020, was summarized in Fig. 1.

### Eligibility criteria

The inclusion of articles complied with the following criteria: (a) study involved proteome profiling and/or quantification based on mass spectrometry techniques; (b) research performed on human aqueous humor or vitreous humor samples; and (c) a group of AMD patients and a healthy control group were valid in research.

Proteomics studies only assessing the effects of specific treatment in AMD patients or only having samples of treated AMD patients will be excluded.

### Quality of evidence

QUADOMICS methodology criteria was used to assess the quality of included studies (Table S1). Two authors evaluated the included studies independently (Figure S1). As a quality evaluation tool, QUADOMICS is appropriative for studies used omics techniques [19, 20].

### Data extraction

Two authors, Hanmu Guo and Jianqing Li, followed fixed protocol to extract data from the eligible studies (Tables 1 and 2): (1) first author; (2) publication year; (3) participants’ characteristics; (4) analytical technique; and (5) altered proteins in AMD patients as measured against controls. Additional information will request from the authors of included studies when necessary.

**Table 1:** Demographic summary of all the studies included in the systematic review and meta-analysis

First author	Year	Type of studies	AMD			Controls			Ref
			**n**	**Age**	**Gender(m/f)**	**n**	**Age**	**Gender(m/f)**	
Batya Rinsky	2021	Observational studies	30	dry AMD: 77.3 ± 10.5, wet AMD: 78.6 ± 7.4,	dry AMD: 10/5 wet AMD: 6/9	20	74.8 ± 7.1	10/10	[21]
Je-Hyun Baek	2018	Observational studies	4	73.5 ± 4.9	0/4	2	73.5 ± 4.9	0/2	[22]
Tae Wan Kim	2012	Observational studies	9	68.9 ± 12.8	5/4	8	73.0 ± 11.4	4/4	[23]
Ching-Yao Tsai	2022	RCT	28	/	/	25	/	/	[24]
Si-Chang Qu	2019	Observational studies	12	80.82 ± 4.17	5/7	12	68.81 ± 18.82	6/6	[25]
Jiaqi Yao	2013	Observational studies	6	56.17 ± 9.28	3/3	6	57.5 ± 2.95	3/3	[26]
Gum-Yong Kang	2014	RCT	14	69.9 ± 7.4	9/5	18	67.3 ± 6.8	5/1	[27]
Hyungwoo Lee	2014	RCT	15	69.8 ± 7.2	8/7	15	70.3 ± 8.0	8/7	[28]
Matthias Nobl	2016	Observational studies	72	76.5 ± 10.0	28/44	16	61.7 ± 11.7	7/9	[29]
Michael Janusz Koss	2014	Observational studies	73	77.8	23/50	15	60.0 ± 16.0	7/8	[30]
Christian Schori	2018	Observational studies	16	dry AMD: 80.3 ± 6.6 wet AMD: 80.8 ± 6.2	5/11	9	70.1 ± 4.1	4/5	[31]

*AMD* Age-related macular degeneration, *m/f* male/female, *Ref* References, *RCT* Randomized controlled trial

**Table 2:** Proteomic studies of AMD and biomarkers discovery using MS-based method in human intraocular fluids

Author (year)	Cohort Information	Sample	Type of Sampling	MS-Based Method	Other Techniques	Quantifi- -cation Method	Depletion/ Enrichment	Altered Proteins	Ref
Batya Rinsky (2021)	15 wet AMD 15 dry AMD 20 controls	Aqueous humor	pooled	label-free	ELISA	label-free	Yes/No	**AMD vs. Controls:** ↑ P10909; P29622; P18065; Q9NPH3; P02753; P00746; P02741; Q9UGM5 ↓ P13726 **dry AMD vs. wet AMD:** ↑P10909; P29622; P13726	[21]
Je-Hyun Baek(2018)	4 dry AMD2 controls	Aqueous humor	individuated	LC–MS/MS	ELISA	SWATH-MS	Yes/No	**AMD vs. Controls:** ↑P02647; P06727; P29622; P51884; O60938; P02647; 29,622; P51884; O60938; P10909 ↓P36980; P10909; P36955; P41222; P01033; P06727; 36,980; P07339; P36955; P41222; P01033	[22]
Tae Wan Kim (2012)	9 AMD 8 controls	Aqueous humor	individuated	LC–ESI–MS/MS	/	MRM	Yes/No	**AMD vs Controls:** ↑ P00450; P36955; P05155; Q15582; P10909; P07339; P01034	[23]
Ching-Yao Tsai (2022)	28 AMD 25 controls	Aqueous humor	pooled	LC–MS/MS	/	MS	No/No	**AMD vs Controls:** ↑P02647; Q6MZU6; Q9UBM4; P10909; P01024; ↓P41222	[24]
Si-Chang Qu (2019)	12 dry AMD 12 controls	Aqueous humor	pooled	LC–MS/MS	ELISA Western Blot	iTRAQ	No/No	**AMD vs Controls:** ↑ P05109; Q14118; P01876 P02763;P06727; P07451 ; P02788; P30838; P01042; P63261; P19823; P02647; P19652; P07225; P25311; P43652 P80748; P00338; P01024; P02750; P02760; P02675; P04196; P00734; P00747; P05546; P61626; P04406; P01775; P00751; P10451; P01023; P35749; P02774; P31025; P01861; P01772; Q9HCQ7; ↓ P49788; O95967; Q9BRK5; P13591; P03950; P23142; P01779; Q13510; P11021; P06309; O00391; Q92563; P61812; Q16270; P00738; Q8N475; P28799; Q96KN2; P30041; Q15113; P51693; P02452; Q06481; P16035; Q16568; O15031; P35555; Q8WXD2; P02766; P22352; Q02809; P06865; Q99435; P01034; P05154; Q12907;P06396; P13645; P07339; P16870; Q9HCB6; P39060; P04264; Q92520; P07477; Q12805; 13,822; P10745; P41222; Q9BU40; O15537	[25]
Jiaqi Yao (2013)	6 wet AMD6 controls	Aqueous humor	pooled	MALDI TOF/TOF MS	ELISA	2-DE	No/No	**AMD vs Controls: ↑**P31025	[26]
Gum-Yong Kang (2014)	26 wet AMD18 Controls	Aqueous humor	pooled	LC–ESI–MS/MS	Western Blot	LC-MRM	No/No	**AMD vs Controls: ↑** P62736; P35579; P07339; P05787; P02533; P34932	[27]
Hyungwoo Lee (2014)	15 AMD15 controls	Aqueous humor	pooled	LC–ESI–MS/MS	Western blot	LC-MRM	No/No	**AMD vs Controls:** ↑P04844	[28]
Matthias Nobl (2016)	72 wet AMD16 controls	Vitreous humor	individuated	LC–MS/MS	ELISA	MS	No/No	**AMD vs Controls:** ↑ P10909; P36955; P41222; P02787; Q8WZ42; P01834; P0CG05; B9A064; P01857; ↓ Q02388; Q9UBM4 P02489; Q5VST9	[29]
Michael Janusz Koss (2014)	73 AMD 15 controls	Vitreous humor	individuated	LC–MS/MS	Western blot	MS	No/No	**AMD vs Controls:** ↑P01871; P02768; P01857; P01008; P01859; P02787; P43652; P04196; P02753; P02647; P02671; P01876; P02765; P02766; P41222; P00738; P22352; P01009	[30]
Christian Schori (2018)	6 dry AMD10 wet AMD9 controls	Vitreous humor	pooled	LC–MS/MS	ELISA	label-free	Yes/Yes	**AMD vs Controls:** ↑ P13716; P07998; Q9H3G5; P06276; P00390; P17948; Q04917; P00352; Q6NS36; P13284; P36222; Q99497; O00187; P15259	[31]

The proteins identified as altered are represented by their entry name as described in UniProt

*AMD* Age-related macular degeneration, *Ref* References, *ELISA* Enzyme-linked immunosorbent assay

### Statistical analysis

GO analysis and KEGG were performed using the DAVID database. The effect size in this meta-analysis was determined to the log2-fold change of measured proteins. Proteins will be included in the meta-analysis if their effect sizes were valid in at least two research studies. There are two types of effect sizes and corresponding significance groups appeared in the studies, one is log(ratio) or ratio correspond with *p* value, the other is group averages correspond with standard deviations, they were all standardized to log2-fold change and corresponding *p* values. Only the statistics of samples from untreated AMD patients will be calculated. The meta-analysis was completed in R (version 4.2.2) combined with R Studio by using the “meta” [32], “metafor” [33], and “dmetar” R packages [34]. String online database and Cytoscape software was utilized to conduct PPI analysis [35].

## Results

### Characteristics of the included articles

The search strategy of included studies in this study was summarized in Fig. 1. As shown in the search results of EMBASE, WOS and PUBMED databases, totally 83 studies were identified after excluding duplicates. 11 studies which fulfilled the eligibility criteria were finally included [21–31]. The demographic characteristics are summarized in Table 1.

### Bias analysis

The quality evaluation results of the studies included in this study are summarized in Figure 1. Items 1(2 studies), 11(5 studies), and 16 (4 studies) were least fulfilled QUADOMICS quality criteria. Most of studies could not assess items 6 and 12 because of insufficient data.

### Analytical technique

AH were dominant sample type used in the eligible studies, in which 8 studies analyzed AH and 3 studies analyzed VH (Table 1) [29–31]. Regarding the MS-based method, 9 out of the 11 studies used LC‒MS/MS analysis [22–25, 27–31]. The other two studies applied MALDI TOF/TOF and intensity-based label-free quantification and were published in 2013 and 2021 [21, 26]. Most studies further applied other techniques to validate protein expression patterns. The most commonly used two approaches were enzyme-linked immunosorbent assay (ELISA) [21, 22, 25, 26, 29, 31], and western blotting [27, 28, 30].

### GO and KEGG analysis

**Fig. 2:**
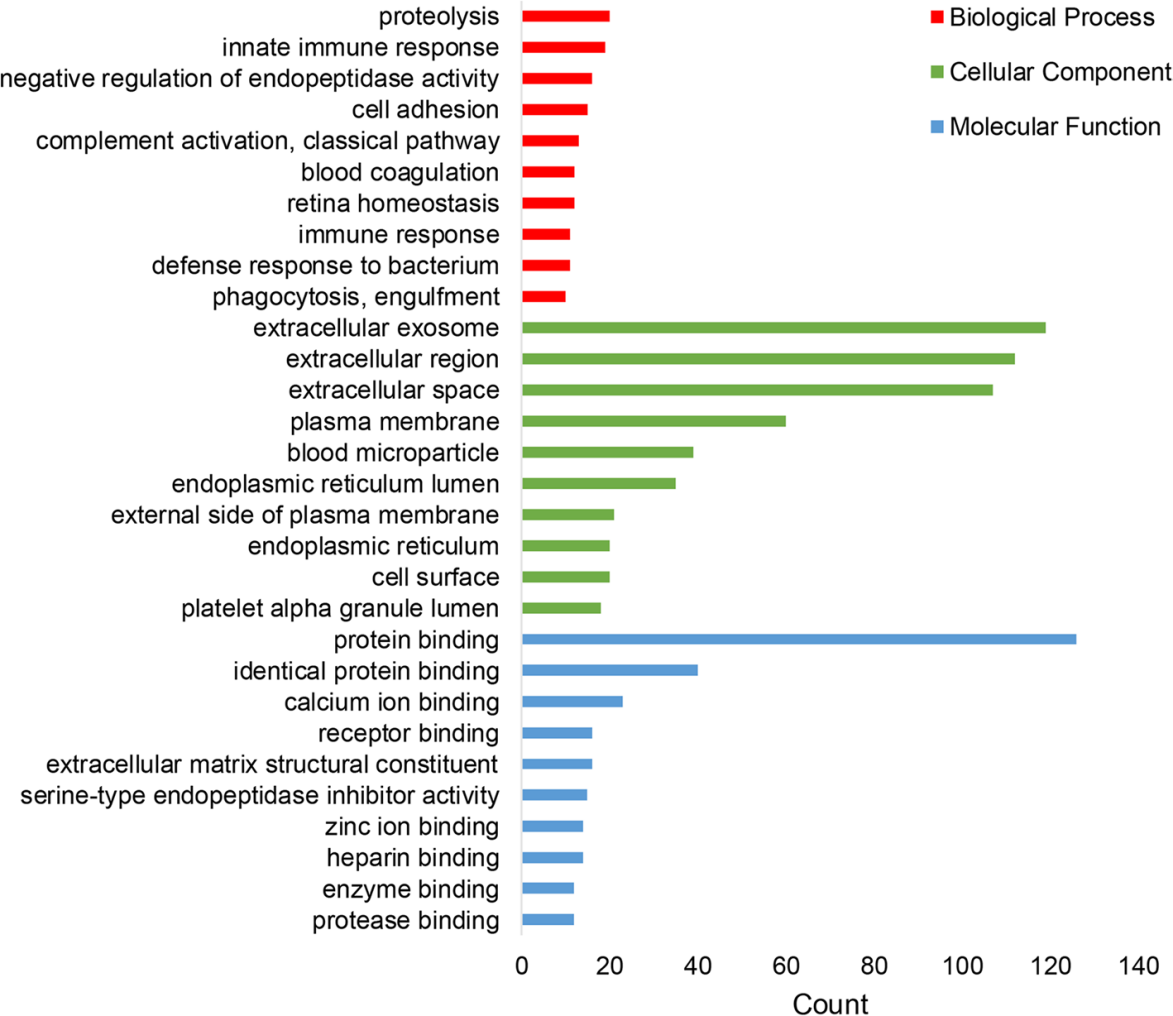
Gene ontology analysis of proteins that were altered in studies were included in the systematic review (top 10 categories based on the protein counts); red represents biological process, green represents cellular component, and blue represents molecular function

GO analysis, which included biological process (BP), cell components (CC) and molecular function (MF), was performed on all the DEPs in the AMD group. Through UNIPROT ACCESSION, 161 proteins were recognized by DAVID Bioinformatics Resources in this ontological analysis, and 10 proteins were not included because no accession number was available. Based on the protein counts, each top 10 categories of BP, CC, and MF were calculated and summarized in Fig. 2. In the BP analysis, the majority of obtained proteins were involved in proteolysis, innate immune response and negative regulation of endopeptidase activity. In terms of CC analysis, most proteins were located in the extracellular exosome, extracellular region, and extracellular space. For the analysis of MF, the results indicated that protein binding, identical protein binding and calcium ion binding were the most important functions.

**Fig. 3:**
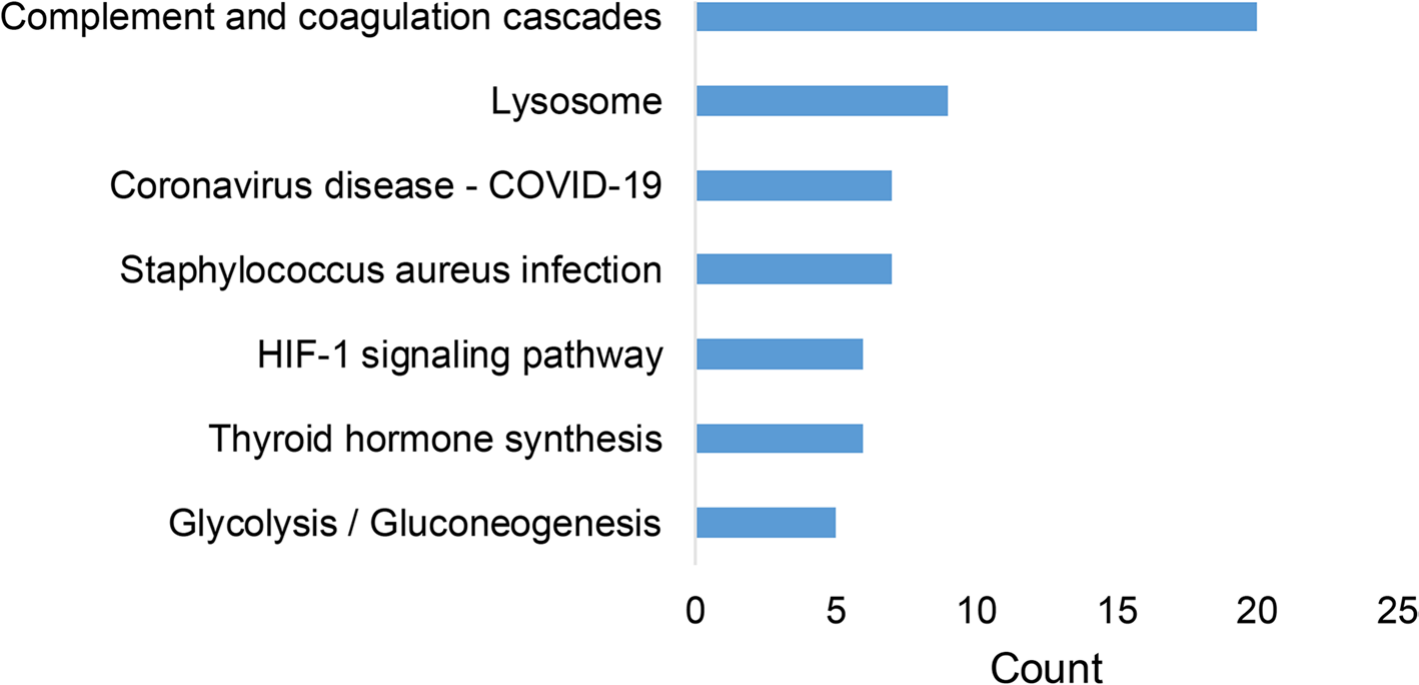
Kyoto encyclopedia of genes and genomes pathway analysis pathway analysis of proteins that were altered in studies was included in the systematic review

KEGG enrichment highlighted seven significantly accumulated pathways involving the DEPs (Fig. 3). Twenty DEPs were enriched in the complement and coagulation cascade pathways, nine DEPs were accumulated in lysosome, and six DEPs were enriched in the hypoxia-inducible factor 1 (HIF-1) signaling pathway.

### Meta-analysis

In all included studies, eight studies [21–28] analyzed AH and three studies analyzed VH [29–31]. The data of DEPs between AMD and control were used to perform the meta-analysis. The effect size (defined as log2-fold change) of proteins will be computed when it could be identified in at least two studies.

**Fig. 4:**
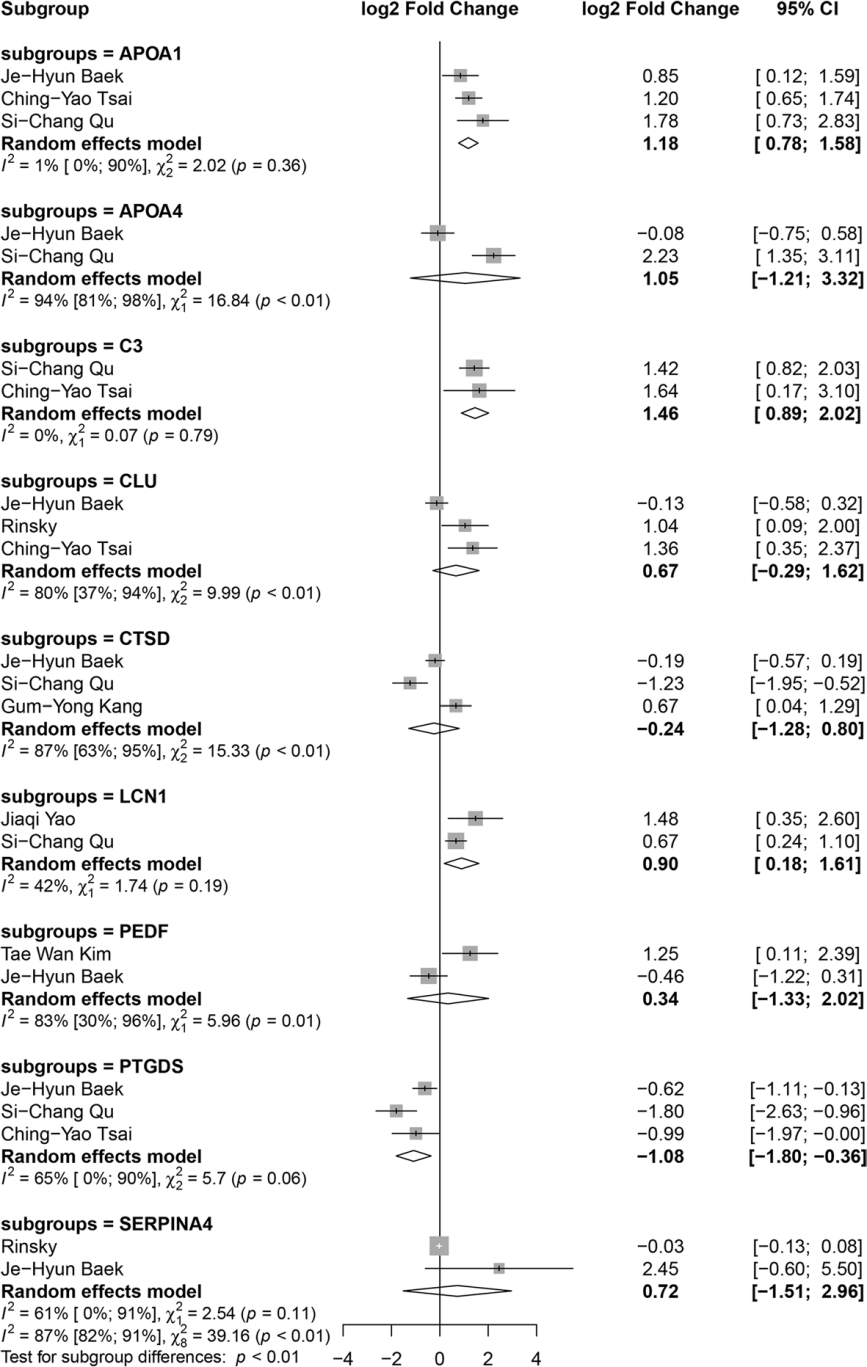
Forest plot from the meta-analysis of proteins identified as altered in AMD vs. control studies analyzed aqueous humor in at least two studies (95% CI, confidence intervals). Squares (whiskers represent 95% CI) indicate the effect sizes of the individual studies. The size of the squares reflects the sample size of each individual study. Diamonds represent summary statistics

The meta-analysis of the DEPs in AH between AMD patients and healthy controls is shown in Fig. 4: apolipoprotein A1 (APOA1, P02647), apolipoprotein A4 (APOA4, P06727), complement C3 (C3, P01024), clusterin (CLU, P10909), cathepsin D (CTSD, P07339), lipocalin-1 (LCN1, P31025), pigment epithelium-derived factor (PEDF, P36955), prostaglandin-H2 D-isomerase (PTGDS or PH2D, P41222) and kallistatin (SERPINA4, P29622) (gene names and accession numbers, respectively). The meta-analysis results suggested increased expression of APOA1, C3, and LCN1 and decreased expression of PTGDS in dry and wet AMD patients compared with healthy control subjects.

**Fig. 5:**
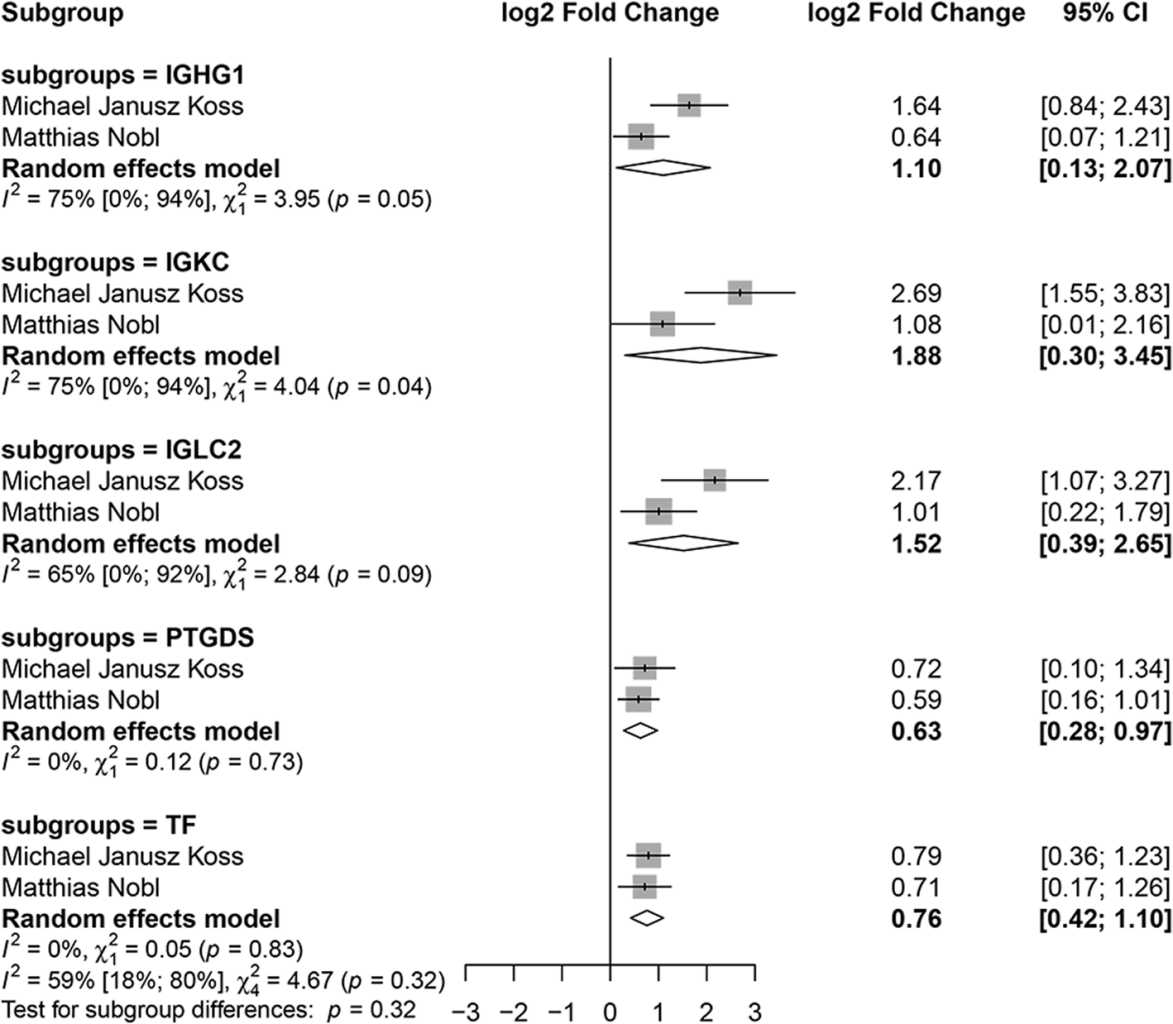
Forest plot from the meta-analysis of proteins identified as altered in AMD vs. control studies analyzed vitreous humor in at least two studies (95% CI, confidence intervals). Squares (whiskers represent 95% CI) indicate the effect sizes of the individual studies. The size of the squares reflects the sample size of each individual study. Diamonds represent summary statistics

The meta-analysis looking at the DEPs in VH between wet AMD patients and healthy controls was performed on five proteins (Fig. 5): Ig gamma − 1 chain C region (IGHG1, P01857), Ig kappa chain C region (IGKC, P01834), Ig lambda − 2 chain C regions (IGLC2, P0DOY2), PTGDS (P41222) and serotransferrin (TF, P02787). All the proteins were more highly expressed in the AMD group.

### PPI analysis

**Fig. 6:**
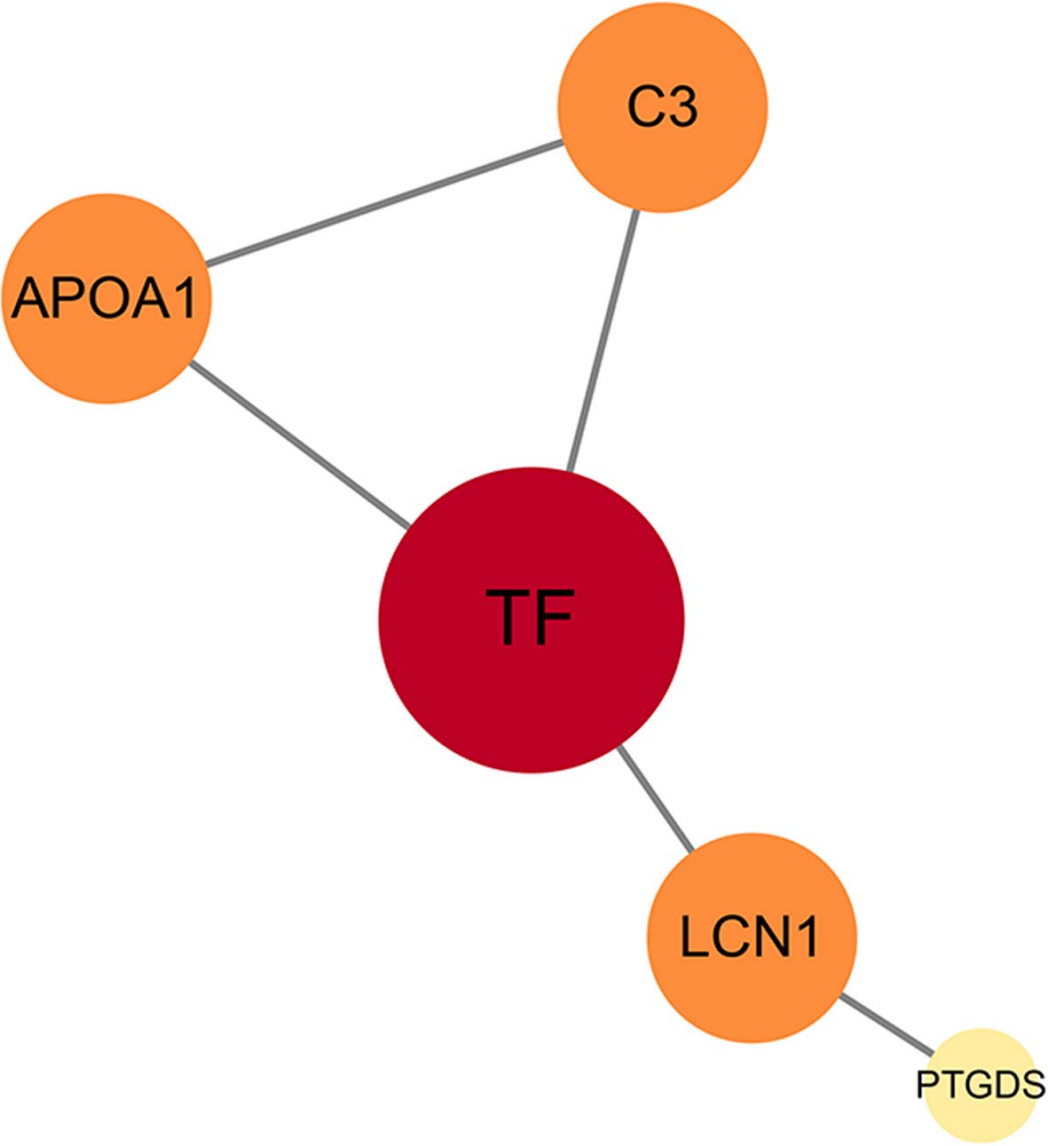
Protein–protein interaction analysis conducted by the String database and Cytoscape software

PPIs was performed by using the STRING database and Cytoscape software in this research [35]. Through the String database, 8 proteins that showed statistical significance in the meta-analysis were filtered into the analysis, finally resulting in 5 nodes and 5 edges (three proteins could not be found in the String database). These statistics are processed by Cytoscape software, and the results are shown in Fig. 6. The figure was created by statistics. The degree dictated the size and color, and the combined score determined the edge size, therefore a low value led to smaller sizes and darker colors than a high value. As shown in the figure, the most significant proteins were TF (P02787), C3 (P01024) and APOA1 (P02647).

## Discussion

To assess protein expression differences between AMD patients and controls, identify biomarkers and related biological pathways of AMD, a comprehensive systematic review and meta-analysis was performed based on mass spectrometry-based proteomics of human intralocular fluids. 161 altered proteins were identified between the AMD and control groups.

GO analysis and KEGG analysis were performed on all the DEPs. GO analysis showed a connection between AMD and the proteolysis, innate immune response, and negative regulation of endopeptidase activity, which mainly occurred in extracellular exosomes, extracellular regions and the extracellular space. In terms of MF, identical protein binding and calcium ion binding were the most important functions. KEGG showed that the most significant pathway was complement and coagulation cascades. The results of GO and KEGG analyses were generally consistent with the known pathogenic mechanism of AMD [9].

The meta-analysis on DEPs in AH found upregulated APOA1, C3, LCN1 and downregulated PTGDS. In separate pooled analysis of these proteins, a consistent trend was shown in both dry and wet AMD patients. The meta-analysis on VH showed IGHG1, IGKC, IGLC2, PTGDS and TF to be overexpressed in wet AMD patients.

PTGDS is downregulated in the AH [22, 24, 25] but upregulated in the VH of AMD patients [29, 30]. PTGDS is an enzyme that converts prostaglandin H2 to prostaglandin D2 and acts as a transport protein for lipophilic substances such as retinoic acid and bilirubin [36]. The relationship between PTGDS and AMD was confirmed by analysis of blood samples [37]. PTGDS is considered a protective factor because it can prevent oxidative stress and apoptosis-related neurodegenerative diseases [38]. The reason for the different trends in PTGDS may be the various degrees of AMD. The retinal pigment epithelium (RPE) is the main source of intraocular PTGDS [39], and mild AMD patients with normal RPE functions may exert protective effects by increasing the level of PTGDS. When RPE functions are damaged to a certain extent in advanced AMD, the level of PTGDS may decrease.

Further study on significant proteins was conducted through PPI analysis. The results indicated that TF, APOA1 and C3 and LCN1 may be the most important proteins, with the former 3 proteins interacting with each other. Based on the results of KEGG analysis, TF was involved in the HIF-1 signaling pathway. C3 was involved in complement and coagulation cascades and Staphylococcus aureus infection pathways. However, APOA1 and LCN1 were not found in the KEGG pathway results.

As iron binding transport proteins, TFs play a role in transporting iron to sites of storage and utilization after absorption or heme degradation. Cell proliferation may also be stimulated by serum transferrin [40, 41]. The serum level of transferrin was found to be higher in the AMD group [42]. Transferrin receptor and variability of its gene might also influence AMD risk [43, 44]. Recent studies discovered that hypoxia may aggravate ferroptosis in RPE cells and then affect the pathophysiology of AMD [45, 46], which provided novel insight into hypoxia, oxidative stress and iron metabolism in AMD pathophysiology. Furthermore, transferrin nonviral gene therapy also showed preliminary effectiveness for the treatment of dry AMD [47]. The above findings indicate that TF may be a promising biomarker of AMD.

APOs are significant proteins for maintaining lipid homeostasis, not only playing an important role in transporting and metabolizing lipids, APOs also relating to regulation of inflammatory and immune response [48, 49]. APOA1 is one of the major components of high-density lipoprotein (HDL) and is considered for regulating levels of free fatty acids in the plasma, involving in metabolism of HDL and triglyceride-rich lipoprotein by the reverse cholesterol transport pathway [50]. Research has shown that elevated HDL cholesterol levels may contribute to formation of drusen in process of AMD [51]. Three meta-analyses have shown elevated APOA1 levels in the AH of AMD patients [22, 24, 25]. Recent studies also showed plasma APOA1 and HDL level may relate to the risk of AMD formation [52–54]. These results indicating that APOA1 may be a potential biomarker in AMD patients, and may participate in formation of AMD. The complement system is considered to play a central role in AMD pathogenesis, and overactivation of the alternative complement pathway is one of the main drivers of diseases and is related to multiple pathogenic factors of AMD, such as inflammation, oxidative stress and lipid accumulation [55, 56]. Before exposing binding sites to the surface of pathogenic cell or other complement components, the C3 protein usually stating biologically inactive [57]. The complement system is frequently activated in many inflammatory diseases, including AMD [55, 58]. As the first line of defense against the innate response, the complement system could recognize and mediate the process of pathogens, debris, and dead cells removal to protect the human organism; hence, therapies targeting complement C3 still need to be considered carefully in AMD patients [55, 59].

It is worth noting that LCN1 also demonstrated importance in PPI. LCN1 is stimulated by oxidative stress, and is considered to be a scavenger of potentially harmful lipid peroxidation products [60]. At present, pathological process of AMD is supposed to relate with oxidative stress, although the specific mechanisms remain unclear [61], and oxidative stress-related genes are also associated with AMD risk [62, 63]. Therefore, LCN1 is considered to have a protective role in the progression of AMD. Except for elevating in the aqueous humor, LCN1 was also found in humans and animals tears in early researches [64]. In a recent study, LCN1 in tears demonstrated its potential as a biomarker in screening diabetic retinopathy [65], which brings expectations for LCN1 to become a biomarker of AMD.

The primary limitation was the heterogeneity among enrolled studies. For example, different studies had different definitions of DEPs, which mainly reflected in the minimum fold change values of proteins to be displayed and the indicators for measuring the accuracy of results (*p* value or adjusted *p* value). Heterogeneity like type of AMD, age or gender was also observed in the cohorts’ characteristics. Limited number of original studies included in each pooled analysis is also a limitation of this study. Although this study included observational and randomized controlled trial (RCT) studies, it did not bring any additional heterogeneity because only statistics before intervention of RCT studies could be included in the meta-analysis, and the criteria for selecting patients were similar in the included RCT and observational studies.

## Conclusions

To sum up, various pathways associated with AMD have been elucidated, including lipid metabolism, the complement system, oxidative stress, inflammation, immunology, iron metabolism and ferroptosis. Four possible biomarkers, TF, APOA1, C3 and LCN1, were found to be significant in the pathogenesis of AMD and need to be further validated. Further studies should be performed to evaluate diagnostic and therapeutic value of these proteins.

### Supplementary Information

**Additional file 1: Table S1.** QUADOMICS criteria to evaluate the quality of the -omics research reports included in a systematic review.

**Additional file 2:** **Figure S1.** QUADOMICS evaluation of the quality of the proteomics studies included in the systematic review.

## Data Availability

The datasets used and/or analysed during the current study are available from the corresponding author on reasonable request.

## Abbreviations

AMD: Age-related macular degeneration
GO: Gene ontology
KEGG: Kyoto encyclopedia of genes and genomes pathway analysis
DEPs: Differentially expressed proteins
PPI: Protein‒protein interaction
GA: Geographic atrophy
nvAMD: Neovascular age-related macular degeneration
AH: Aqueous humor
VH: Vitreous humor
LC‒MS/MS: Liquid chromatography coupled to tandem mass spectrometry
WOS: Web of Science
ELISA: Enzyme-linked immunosorbent assay
BP: Biological process
CC: Cell components
MF: Molecular function
MS: Mass spectrometry
APOA1: Apolipoprotein A1
APOA4: Apolipoprotein A4
C3: Complement C3
CLU: Clusterin
CTSD: Cathepsin D
LCN1: Lipocalin-1
PEDF: Pigment epithelium-derived factor
PTGDS: Prostaglandin-H2 D-isomerase
SERPINA4: Kallistatin
HIF-1: Hypoxia-inducible factor 1
IGHG1: Ig gamma − 1 chain C region
IGKC: Ig kappa chain C region
IGLC2: Ig lambda − 2 chain C regions
TF: Serotransferrin
HDL: High-density lipoprotein
RCT: Randomized controlled trial
