# Supplementary material for: Systematic review and meta-analysis of mass spectrometry proteomics applied to ocular fluids to assess potential biomarkers of age-related macular degeneration
Source: BMC Ophthalmol. 2023 Dec 12;23:507. doi: 10.1186/s12886-023-03237-0 (PMC10717315; doi:10.1186/s12886-023-03237-0)
Supplement: Supplementary file 1 — Additional file 1: Table S1. QUADOMICS criteria to evaluate the quality of the -omics research reports included in a systematic review. [file 12886_2023_3237_MOESM1_ESM.docx]

| Item |
| --- |
|  |
| Study Phase: Phase 1, 2, 3, 4  1. Were selection criteria clearly described? |
| 2. Was the spectrum of patients representative of patients who will receive the test in practice? * |
| 3. Was the type of sample fully described? |
| 4. Were the procedures and timing of biological sample collection with respect to clinical factors described with enough detail? |
| 4.1. Clinical and physiological factors  4.2. Diagnostic and treatment procedures. |
| 5. Were handling and pre-analytical procedures reported in sufficient detail and similar for the whole sample? And, if differences in procedures were reported, was their effect on the results assessed? |
| 6. Is the time period between the reference standard and the index test short enough to reasonably guarantee that the target condition did not change between the two tests? |
| 7. Is the reference standard likely to correctly classify the target condition? |
| 8. Did the whole sample or a random selection of the sample receive verification using a reference standard of diagnosis?  9. Did patients receive the same reference standard regardless of the result of the index test?  10. Was the execution of the index test described in sufficient detail to permit replication of the test?  11. Was the execution of the reference standard described in sufficient detail to permit its replication?  12. Were the index test results interpreted without knowledge of the results of the reference standard? |
| 13. Were the reference standard results interpreted without knowledge of the results of the index test?  14. Were the same clinical data available when test results were interpreted as would be available when the test is used in practice? *  15. Were uninterpretable/intermediate test results reported?  16. Is it likely that the presence of overfitting was avoided? |

TABLE. s1. QUADOMICS criteria to evaluate the quality of the -omics research reports included in a systematic review

*Applicable only to phase 3 or 4 studies
