# Supplementary figures and images for: Systematic review and meta-analysis of mass spectrometry proteomics applied to ocular fluids to assess potential biomarkers of age-related macular degeneration
Source: BMC Ophthalmol. 2023 Dec 12;23:507. doi: 10.1186/s12886-023-03237-0 (PMC10717315; doi:10.1186/s12886-023-03237-0)

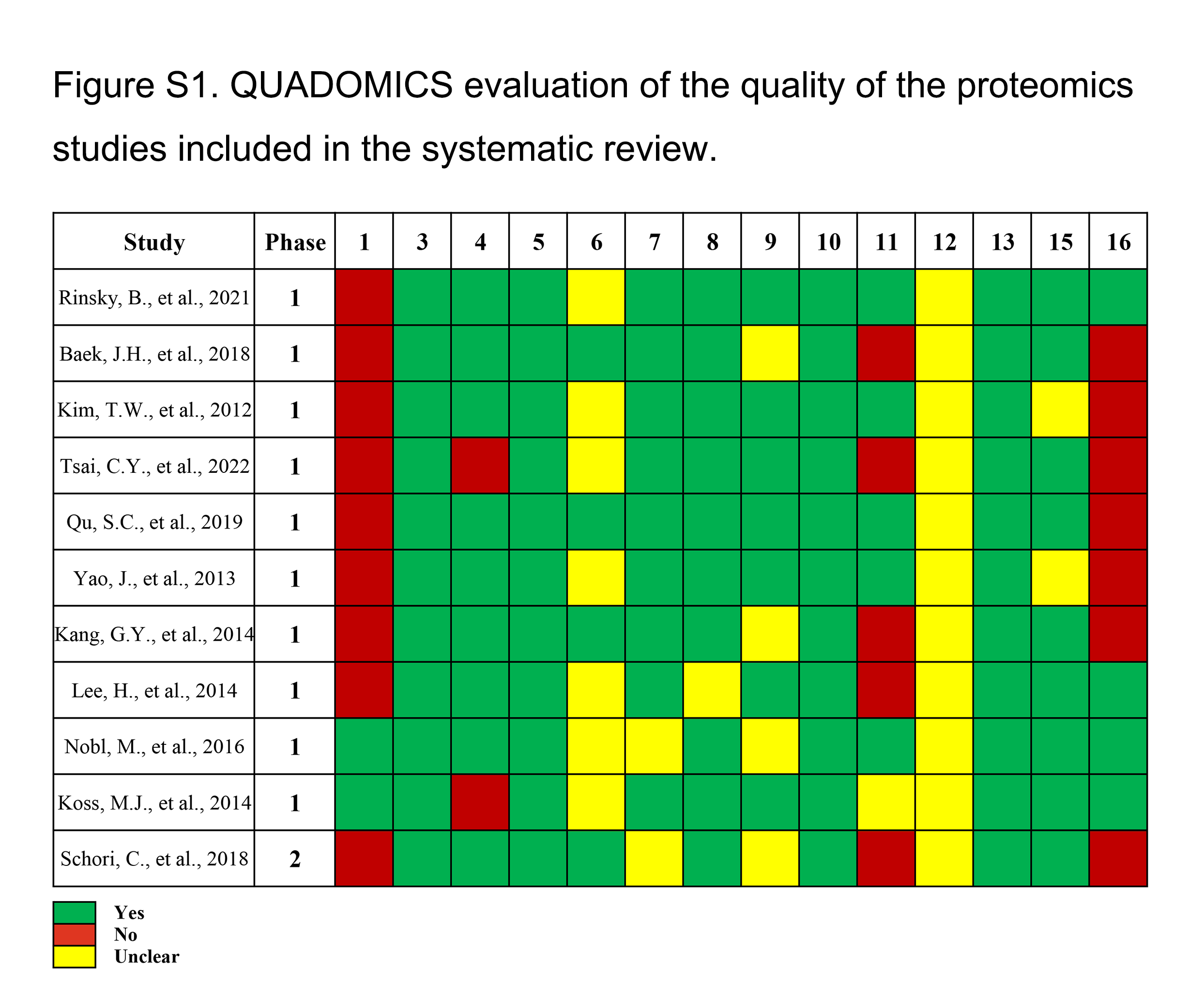

Supplement: Supplementary file 2 — Additional file 2: Figure S1. QUADOMICS evaluation of the quality of the proteomics studies included in the systematic review. [file 12886_2023_3237_MOESM2_ESM.tif]
